# Lanthanide Contraction in *Ln*F_3_ (*Ln* = Ce-Lu) and Its Chemical and Structural Consequences: Part 1: Location of YF_3_ in the *Ln*F_3_ Series According to Its Chemical and Structural Characteristics

**DOI:** 10.3390/ijms242317013

**Published:** 2023-11-30

**Authors:** Boris P. Sobolev, Elena A. Sulyanova

**Affiliations:** Shubnikov Institute of Crystallography, Federal Scientific Research Centre “Crystallography and Photonics”, Russian Academy of Sciences, Leninskiy Prospekt 59, 119333 Moscow, Russia; sobolev.b@crys.ras.ru

**Keywords:** lanthanide contraction, rare earth elements, lanthanides, yttrium trifluoride, heats of phase transformations, polymorphism, phase diagrams

## Abstract

A *lanthanide contraction* **(LC)** of 14 *lanthanides* (*Ln*) from _58_Ce to _71_Lu consists of the interaction of *Ln* nucleus with *4f*-electrons. *Rare earth elements* (REEs—*R*) include Sc, Y, La, and 14 *Ln*. They are located in 4–6th periods of the subgroup of group III. The electronic structure divides *R* into short (*d*- Sc, Y, La) and long (14 *f*-elements Ce-Lu) homologous series. The most important chemical consequence of LC is the creation of a new conglomerate of 16 *R*F_3_ by mixing fluorides of *d*- (Y, La) and *f*-elements. This determines the location of YF_3_ among *Ln*F_3_. The location of YF_3_ depends on the structural (*formula volumes*—**V_form_**) and thermochemical (temperatures and heats of phase transformations, phase diagrams) properties. The location of YF_3_ between HoF_3_ and ErF_3_ was determined by V_form_ at a *standard pressure* (***P*_st_**) and *temperature* (***T*_st_**). The location of YF_3_ according to *heats of phase transformations* Δ**H*_fus_*** and Δ**H_trans_** is in a dimorphic *structural subgroup* (**SSGr**) ***D*** (*Ln =* Er-Lu), but without the exact “*pseudo* Z_Y_”. According to the *temperatures of phase transformations* (***T***_trans_) in *Ln*F_3_ (*Ln* = Dy-Lu), YF_3_ is located in the SSGr ***D*** between ErF_3_ and TmF_3_. The ErF_3_-YF_3_ and YF_3_-TmF_3_ phase diagrams show it to be between ErF_3_ and TmF_3_. The crystals of five ***β***-*Ln*F_3_ (*Ln* = Ho-Lu) and ***β***-YF_3_ were obtained in identical conditions and their crystal structures were studied. V_form_ (at ***P***_st_ and ***T***_st_) with “*pseudo*” *atomic number* **Z**_Y_ = 67.42 was calculated from the unit cell parameters, which were defined with ±5 × 10^−4^ Å accuracy. It determines the location of YF_3_ between HoF_3_ and ErF_3_.

## 1. Introduction

This study opens up a series of investigations on LCs and their chemical and structural consequences in homologous series of LaF_3_ and 14 *Ln*F_3_. The (La,*Ln*)F_3_ series differs from the homologous series of *Ln* compounds by characteristics that make it unique for the precise study of LCs. The formula for the (La,*Ln*)F_3_ series is simple. It determines the qualitative and quantitative chemical compositions that are favorable for the LC study. Fluorine anion has low atomic mass (19). High atomic masses of *Ln* lead at the beginning of the series in CeF_3_ to 71% and at the end of LuF_3_ to ~75% of the mass of compressible *Ln*^3+^. The LC in *Ln*F_3_ has an extremely high “sensitivity” to volumetric changes when filling the 4*f*-orbital.

Despite the difference in the electronic structure, according to IUPAC recommendation, La is classified as *Ln*. This disadvantage of REE classification was noted by IUPAC as a project to clarify the location of La (2015). In this study, the formula (La,*Ln*)F_3_ is used when discussing issues related to LCs. This formula is not equivalent to a structural formula that isolates chemical elements in equivalent structural positions. The purpose of the (La,*Ln*) designation is to separate 14 *Ln* (4*f*-elements), which are exposed to a LC, from La, which has no *f*-electrons. When LC is not discussed, the *R* abbreviations for Y, La, and 14 *Ln* are used.

Owing to the extreme electronegativity of fluorine 4.0 according to [1] (after helium and neon), *R*F_3_ compounds have a high degree of a chemical bond ionicity; therefore, they are characterized by high melting points (***T***_fus_). They crystallize from the melt in the form of large crystalline blocks, which are suitable for structural studies. All *R*F_3_ are chemically resistant, except SmF_3_, EuF_3,_ and YbF_3_. They were subjected to partial reduction under the conditions of an experiment on crystal growth and the study of phase diagrams with their participation [2,3,4,5,6,7].

The location of YF_3_ in the *Ln*F_3_ series is determined not by its Z = 39 but by the structural and chemical characteristics of _39_YF_3_, independent of LC. Therefore, this location is ambiguous. According to the literature, the location of YF_3_ depends on its structural (unit cell parameters, formula volumes) or thermochemical (temperature, heat of phase transformation, phase diagrams) properties. In this study, a critical analysis of the structural and thermal characteristics of YF_3_ is performed according to the data of various research groups and our own precise structural data.

The term LC was first used in [8], and the name was given to the family of 14 *Ln* (“similar to lanthanum”). The *Ln*^3+^ ionic radius in REE oxides was chosen as a characteristic of LC. According to [8], the *radius* of *R*^3+^ (***r***_+_) decreases from La^3+^ to Lu^3+^ by ~15%. These data have been included in the educational literature and are still being cited. The radii were obtained on an incomplete REE series. Owing to the unresolved problem of REE separation in those years, the purity of some oxides by the main component had reached 50% at. Historically, ***r***_+_ in *R*_2_O_3_ has become the first example of a comparative study of LC.

The compression of *Ln*^3+^ ions is expressed as an integral effect, that is, a change in the volume of a crystal. To compare non-cubic crystals, which are all *R*F_3_, it is convenient to express changes in the unit cell parameters using V_form_. The ultimate goal of precise LC studies is to obtain the dependence of ***r***_+_ on Z for ***P***_st_ and ***T***_st_: ***r****_Ln_* = ***f*_0_**(Z).

A change in the state parameter ***T*** > ***T***_st_ causes structural changes in the *R*F_3_ series: *polymorphic* (**PolTr**) and *morphotropic transformations*. Both transformations proceed with abrupt changes in V_form_. The *R*F_3_ PolTr exhibit a *density anomaly*. The high-temperature form is denser (usually opposite). Materials that expand after PolTr at heating are called *materials with negative thermal expansion based on a phase transition-type mechanism* [9]. V_form_ changes with Z according to different dependencies: for the LaF_3_ type (hereafter ***t***-) by V*_t_*_-_ = ***f*_1_**(Z), and for the ***β***-YF_3_ type (hereinafter ***β***-) by V***_β_***_-_ = ***f*_2_**(Z).

The difference in the electronic structure requires dividing the REE into short Sc, Y, La (*d*-elements), and long (14 *f*-elements) homologous series. In the short series, _21_Sc→_39_Y→_57_La, ***r***_+_ increases in group III in the ΔZ = 18 interval. In the long series, a LC phenomenon, which is unique to the periodic table of elements, is realized. The LC of the elements of period 6 (*Ln* = _58_Ce-_71_Lu) is a result of an interaction in the “*Ln* nucleus—4*f*-electrons” atomic subsystem. The minimum “period” of the long series is ΔZ = 1. This corresponds to the transition between neighboring *Ln*F_3_. At the same time, the size of *Ln*^3+^ cation decreases significantly with increasing Z due to LC.

*d*-REE La occupies a dual position. Having no *f*-electrons, La heads the *Ln* family, giving it a name. The IUPAC assignment of La to *Ln* is caused by the neighborhood of _57_La in the 6th period with _58_Ce at ΔZ = 1.

Yttrium has no *f*-electrons, and its ***r***_+_ is independent of LC. The indirect effect of LC on the rapprochement of YF_3_ structural and chemical properties with *Ln*F_3_ properties is due to the influence of LC on *Ln*^3+^. This is expressed as a decrease in ***r***_+_ with an increase in Z.

The location of LaF_3_ at the beginning of the *Ln*F_3_ series does not raise any questions. The Periodic Table of elements places it there by Z(La) = 57. This location before _58_CeF_3_ is also justified by the structural and chemical proximity of LaF_3_ and CeF_3_ (ΔZ = 1).

A question arises about the location of YF_3_ in the *Ln*F_3_ series. Unlike the unambiguous location of _57_LaF_3_, the location of _39_YF_3_ is determined not by its Z = 39, but by chemical and structural characteristics. The certainty of chemical characteristics is much less than the structural and absolute (Z) ones. In the literature there are several proposed locations of YF_3_.

The structural classification of 16 *R*F_3_ (without ScF_3_) homologous series and the chemical classification of the phase diagrams of 120 (without ScF_3_) *R*F_3_-*R*’F_3_ systems depend on the place of YF_3_ in the *Ln*F_3_ series. The *R*F_3_ structural classification is the first level of a chemical classification of *R*F_3_-*R*’F_3_ systems.

To determine the location of YF_3_ in the *Ln*F_3_ series, only precise information on LC in *Ln*F_3_ is suitable. Two conditions ensure its receipt.

The *first* is the individuality of the homologous series of compounds. In this series of messages, for the first time, LC studies were performed on one (long) homologous series of REE fluorides: LaF_3_ and 14 *Ln*F_3_. The high degree of bond ionicity and simple formula of the series give the absence of “blurring” of the boundaries of the change in structural types over the *Ln* series (for fixed ***P*** and ***T***). This “blurring” accompanies all structural classifications of *Ln* compounds using their large arrays. The choice of compounds for arrays usually does not take into account differences in their chemical bonds. Arrays of compounds are heterogeneous in the chemical bonds. The conclusions of the most fundamental monograph [10] with an overview of 400 homologous series of REE compounds will be discussed in detail in the next message.

The general conclusion in [10] after processing a unique volume of data on LCs in the series of *Ln* compounds is categorical. “It is impossible to obtain an ideal system of REE ionic radii by averaging interatomic distances taken from a large number of structural definitions”. “Blurring” the several atomic numbers of the boundaries of “areas of crystal and chemical instability” [10] does not allow us to determine the subtle features of the LC evolution in the *Ln* series. These data are suitable neither for a precise study of the evolution of LC nor for constructing a *specialized* (**Spec-zd**) *empirical* (**Emp**) *system of ionic radii* (**SIR**) for *R*F_3_.

The *second condition* is the completeness of the *Ln*F_3_ structural characteristics used to describe the changes in V_form_ during LC. The state of structural knowledge of *R*F_3_ is currently unsatisfactory both in terms of the number of studied compounds (about half) and in terms of the purity of the reagents used, especially in early works.

Structural studies of *R*F_3_ stretched over the years 1929–2023. During this period, significant changes occurred in the composition of *R*F_3_ crystals. It can be said that in the works of 30–70s, the purity of crystals by impurities of neighboring REE was not high. And only in 1971, YF_3_, LaF_3_, PrF_3_, NdF_3_, GdF_3_, HoF_3_, and LuF_3_ were published, with total REE content of 35–125 ppmw and with other cations content of 30–55 ppmw [11]. Cationic and anionic (oxygen) impurities are difficult to control. The structural data of *R*F_3_ distorted by them are unsuitable for precise studies of LC and the construction of the *Spec-zd Emp* SIR for (*R*,*Ln*)F_3_.

In this series of studies, the crystal structures of all members of the (*R*,*Ln*)F_3_ series are investigated. The structures of HoF_3_, ErF_3_, TmF_3_, YbF_3_, LuF_3_, and YF_3_ are discussed in the present study. The short *quasi-system* (**QS**) [12] “from HoF_3_ to LuF_3_” is used to obtain “*pseudo* Z_Y_”. The location of YF_3_ is determined to be between HoF_3_ and ErF_3_ based on the value of V_form_. The second message presents the structural data of ***t*-***R*F_3_ with *R* = La-Nd of the LaF_3_ type, “*pseudo **t***-SmF_3_”, and ***β*-***Ln*F_3_ (*Ln* = Sm-Dy) of the ***β***-YF_3_ type. The empirical structural data obtained for cations and fluorine anion will become the basis for the *Spec-zd Emp* SIR for Y^3+^, La^3+^, and 14 *Ln*^3+^ for *R*F_3_.

The *aim of this study* is to clarify the most extensive indirect chemical consequence of LC, which is the location of fluoride of the *d*-element YF_3_ in a series of 4*f*-elements *Ln*F_3_.

## 2. Results

### 2.1. Obtaining YF_3_ and LnF_3_ with Ln = Ho-Lu for Structural Studies

*X-ray diffraction* (**XRD**) analysis of YF_3_ and *Ln*F_3_ with *Ln* = Ho-Lu was performed on the samples prepared using modern technology for the synthesis of crystals prone to pyrohydrolysis REE fluorides.

The *R*F_3_ reagents were prepared at the experimental plant (town Pyshma) of the Government Institute of Rare Metals (GIREDMET, Moscow, Russia). *R*F_3_ were melted and fluorinated to purify oxygen impurities. Oxygen content of 0.005–0.08 wt. % has been achieved (determination by vacuum melting) [13,14]. Differential thermal analysis used for the study of phase diagrams provided control over the oxygen impurity content in *R*F_3_ (in the form of an isomorphic admixture of *R*F_3−2x_O_x_ oxyfluorides) at an acceptable level [15].

The purity of the reagents by the main component was 99.9 wt. %. Each reagent was certified for REE impurities based on X-ray fluorescence analysis. The typical impurity composition of YF_3_ produced by Pyshma, according to [16], is listed in Table 1.

After fluorination, the samples consisted of large crystalline blocks suitable for structural analysis.

### 2.2. X-ray Diffraction Study of **β**-YF_3_ and Five **β**-LnF_3_ (Ln = Ho-Lu)

To accurately determine the position of YF_3_ in the *Ln*F_3_ series using structural characteristics the structures of ***β***-YF_3_ and five ***β***-*Ln*F_3_ (*Ln* = Ho-Lu) were studied.

XRD study were performed using modern equipment for structural analysis under comparable experimental conditions. Only the combination of these factors, which were absent earlier when creating *universal* (***Univ***) SIRs, provides high accuracy in describing the evolution of LC in the *Ln*F_3_ series and calculating comparable values of ***r***_+_ for the *Spec-zd Emp* SIR for *R*F_3_.

Single crystal XRD study of ***β***-YF_3_ and ***β***-*Ln*F_3_ (*Ln* = Ho-Lu) was performed at 293 K using an XtaLAB Synergy-DW (Rigaku Oxford Diffraction, Japan-UK-Poland) diffractometer with an Ag-anode X-ray tube. The data were processed using the CrysAlisPro version 171.42.72 (Rigaku Oxford Diffraction, Japan-UK-Poland) software package.

Powder XRD analysis was performed at 293 K with a Rigaku MiniFlex 600 Bragg-Brentano diffractometer using a Cu-anode X-ray tube within the range of 2θ = 10–100° and with a 2θ step size of 0.02°. A NIST 640e standard (Si) sample was added to the sample to determine the 2θ correction. The details of the XRD experiments are listed in Table 2.

The JANA2020 program [17] was used for the structure solution and refinement. The structures were refined within *Pnma* sp. gr. An isotropic extinction correction was introduced into the fitted models according to the Becker–Coppens formalism [18]. The Wickoff positions (W.p.) [19], coordinates, site occupancy factors, and equivalent atomic displacement parameters for ***β***-YF_3_ and ***β***-*Ln*F_3_ (*Ln* = Ho-Lu) at 293 K are listed in Table 3.

## 3. Discussion

### 3.1. Determination of “Pseudo Z_Y_” According to Literature Data

The *first group* of structural properties is the type of structure and unit cell parameters. The most precise (±0.0005 Å) measurements of the *R*F_3_ unit cell parameters in a Guinier chamber were used [11,20]. The YF_3_ unit cell parameters [11] differs from that of [20]. The reason for this difference is unknown.

The *second group* includes the thermal properties: the temperatures (***T***_fus_) and enthalpies Δ***H***_fus_ of melting and ***T***_trans_ and Δ***H***_trans_ of the PolTrs. These characteristics determine the location of YF_3_ in the *Ln*F_3_ series for condensed systems with ***P***_st_ and ***T***_st_. The condensed (at ***P***_st_) single-component (Z = const) systems include YF_3_, LaF_3_, and all *Ln*F_3_ except volatile (at heating) ScF_3_ [21,22]. In such systems, PolTr is invariant and proceeds “at a point” ***T*** = ***T***_trans_ = const.

The ***T***_trans_ were taken from a review [23] as the average of those published before 2003 for fluorides of *f*-elements. There is no ***T***_trans_ for YF_3_ among them.

Δ***H***_fus_ and Δ***H***_trans_ for all *R*F_3_ were cited from the fundamental research of one group of authors [11,24]. Synthesis and analysis of the basic and impurity *R*F_3_ compositions and purification from oxygen (control by analysis) were performed at Iowa State University, USA.

Phase diagrams of the two-component *R*F_3_-*R*’F_3_ systems were used for the first time to clarify the location of YF_3_. These systems were studied by one group [25]. The phase diagrams of the *R*F_3_-*R*’F_3_ systems contain data on the ***T***_trans_ of the components. The location of YF_3_ in the short QS [12] depends on the thermal properties of the *Ln*F_3_ components that form this QS.

### 3.2. Location of YF_3_ in the LnF_3_ Series in Terms of the Structural Types and Unit Cell Parameters of 16 RF_3_ (without ScF_3_) at P_st_ and T_st_

The generally accepted scheme for the change of ***T***_trans_ and crystal structures of 17 *R*F_3_ was formed by the mid-80s of the last century by three scientific groups: the USA, Russia, and Germany. However, six outdated schemes were also cited. This forces us to repeat up-to-date ideas about *R*F_3_ structures.

At ***T*** < ***T***_fus_ *R*F_3_ crystallize in four structural types: (1) ScF_3_ (ReO_3_ type), (2) ***β***-YF_3_ (***β***-), (3) LaF_3_ (***t***-), and (4) ***α***-YF_3_ (***α***-). The ***α***-type is stable close to its melting point and was observed in situ only.

The structural classification of *R*F_3_ (without ScF_3_) based on the type of structure and presence (absence) of polymorphism is presented in Table 4. Four SSGr were allocated. The members of the SSGr ***A*** (LaF_3_, CeF_3_, PrF_3_, and NdF_3_) and ***C*** (TbF_3_, DyF_3_, and HoF_3_) are monomorphic and have ***t***- and ***β***- structural types up to the melting point, respectively. The members of the dimorphic SSGr ***B***—PmF_3_, SmF_3_, EuF_3_, and GdF_3_—have ***t***- (high-temperature) and ***β***- (low-temperature) structural types. The members of the dimorphic SSGr ***D*** (ErF_3_, TmF_3_, YbF_3_, and LuF_3_) have ***α***- (high temperature) and ***β***- (low temperature) structural types.

The ***β***-YF_3_ unit cell parameters according to [11,20,24,26] together with V_form_s calculated from them at ***T***_st_ and ***P***_st_ are listed in Table 5. In the right column, the *Ln*F_3_-*Ln*’F_3_ systems are provided, in which V_form_ of YF_3_ is located between the V_form_s of components.

The V_form_s of YF_3_ according to [11,26] are close to each other. They locate YF_3_ between HoF_3_ and ErF_3_. According to [20] YF_3_ is located between DyF_3_ and HoF_3_. This is contradicted by the only form of ***β***- in both fluorides before ***T***_fus_. These data were excluded.

### 3.3. Location of YF_3_ in the LnF_3_ Series in Terms of T_fus_ and ΔH_trans_

A review of ***T***_trans_ in (La,*Ln*)F_3_ (without ScF_3_ andYF_3_) was done [23]. The ***T***_trans_ for each (La,*Ln*)F_3_ was obtained with an accuracy of ±3 °C. The limitation [23] is in the exclusion of YF_3_ from the list as a fluoride of *d*-element. For YF_3_, we adopted ***T***_trans_ from [11].

**Figure 1:**
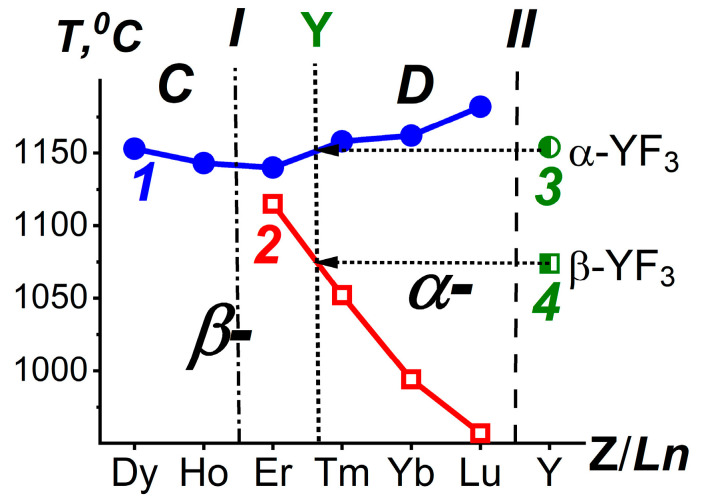
The location of YF_3_ in the *Ln*F_3_ series based on the average ***T***_fus_ (1), ***T***_trans_ (2) of *Ln*F_3_ with *Ln* = Dy-Lu and YF_3_ (3, 4). Curve 1—***T***_fus_ of *Ln*F_3_ with *Ln* = Dy-Lu (blue solid circles), curve 2—***T***_trans_ of *Ln*F_3_ with *Ln* = Er-Lu (red open rectangles). The ***T***_fus_ (3) and ***T***_trans_ (4) of YF_3_ are shown by green semi-open icons. Vertical I is the boundary between structural subgroups ***C*** (*Ln* = Tb-Ho) and ***D*** (*Ln* = Er-Lu). Vertical II separates _39_Y from the Z-scale for *Ln*.

The blue solid circles in Figure 1 show the ***T***_fus_ (curve 1) and red open rectangles (curve 2) ***T***_trans_ of *Ln*F_3_ in the short QS “from DyF_3_ to LuF_3_”. The fields of structural modifications are designated as ***β***- and ***α***-. The border between ***C*** and ***D*** SSGrs is marked with a dash-dotted vertical I.

The ***T***_fus_ and ***T***_trans_ of YF_3_ are shown by green semi-open icons. These are separated from the data for *Ln*F_3_ series by vertical II. The arrows to the left of these points intersect curves 1 and 2 near Z = 69, corresponding to _69_Tm. The intersections are shown as Y-vertical. According to ***T***_trans_ in *Ln*F_3_ (*Ln* = Dy-Lu) [23], YF_3_ is located between ErF_3_ and TmF_3_. This is consistent with the location of YF_3_ in the SSGr ***D***.

**Figure 2:**
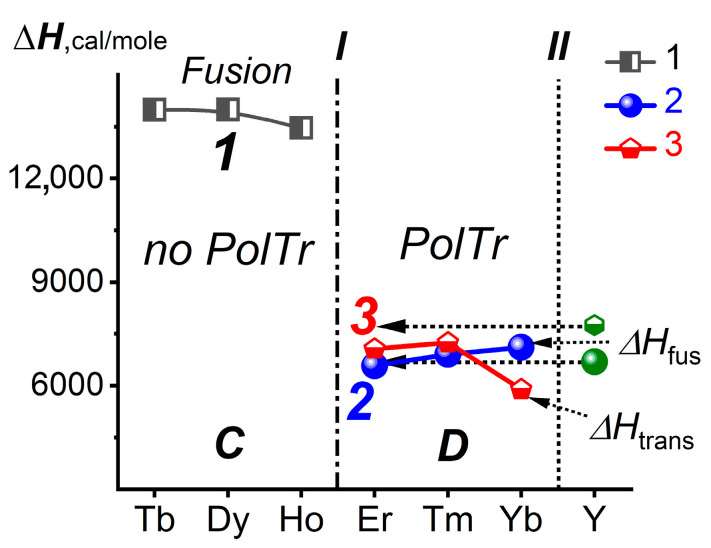
Δ***H***_fus_ and Δ***H***_trans_ for *Ln*F_3_ (*Ln* = Tb-Yb) and YF_3_. Curve 1—Δ***H***_fus_ for *Ln*F_3_ with *Ln*=Tb-Ho (grey semi-open rectangles), curve 2—Δ***H***_fus_ for *Ln*F_3_ with *Ln* = Er-Yb (blue solid circles), curve 3—Δ***H***_trans_ for *Ln*F_3_ with *Ln* = Er-Yb (red semi-open rhombs). Δ***H***_fus_ and Δ***H***_trans_ of YF_3_ are shown by green icons. Vertical I is the boundary between structural subgroups ***C*** (*Ln* = Tb-Ho) and ***D*** (*Ln* = Er-Lu). Vertical II separates _39_Y from the Z-scale for *Ln*.

The Δ***H***_fus_ for *Ln*F_3_ with *Ln* = Tb-Yb are shown in Figure 2 by curves 1 (grey semi-open rectangles) and 2 (blue solid circles). Curve 3 (red semi-open rhombs) corresponds to Δ***H***_trans_ for *Ln*F_3_ of the dimorphic SSGr ***D*** (ErF_3_-YbF_3_) at ***P***_st_ [11,24].

The Δ***H***_fus_ and Δ***H***_trans_ of dimorphic YF_3_ are shown in Figure 2 by green icons. The values of the Δ***H***_fus_ and Δ***H***_trans_ of YF_3_ are projected into the Z region corresponding to ErF_3_ without intersecting curves 2 and 3, respectively. According to Δ***H***_fus_ and Δ***H***_trans_, YF_3_ is located in the SSGr ***D***. However, the location of YF_3_ cannot be precisely determined because of the proximity of the Δ***H*** values. Despite the high accuracy of thermophysical measurements [11,24], they do not contain strict information regarding the location of YF_3_.

### 3.4. Location of YF_3_ Based on the YF_3_-LnF_3_ Phase Diagrams at P = P_st_, T > T_st_

**Figure 3:**
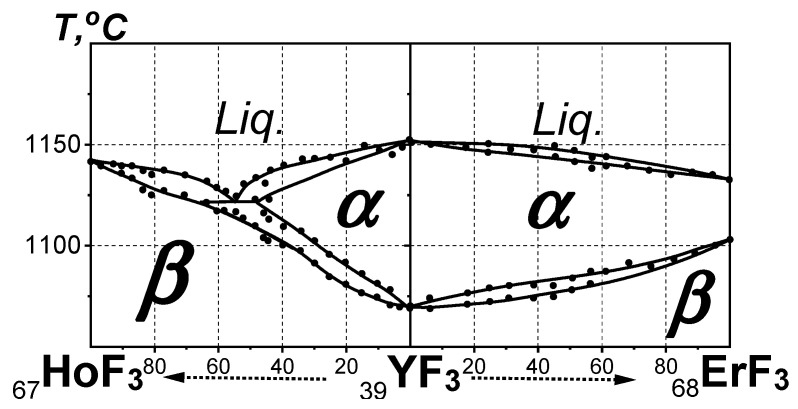
The phase diagram of the short composite “HoF_3_-YF_3_-ErF_3_” QS (***P*** = ***P***_st_; ***T*** > ***T***_st_). Black solid circles correspond to experimental data.

The possible location of YF_3_ on the basis of phase diagrams was obtained based on the location of YF_3_ between HoF_3_ and ErF_3_ determined from unit cell parameters and V_form_s. The composite short “HoF_3_-YF_3_-ErF_3_” QS, including these fluorides (Figure 3), was analyzed.

The phase diagrams of the HoF_3_-YF_3_ and YF_3_-ErF_3_ systems [25] are not included in the full “from LaF_3_ to LuF_3_” QS [12]. The short “HoF_3_-YF_3_-ErF_3_” QS is called *composite* to emphasize the violation of the ΔZ = 1 condition for (*R*,*Ln*)F_3_ sequence in the true QS by “inserting” a component— a fluoride of the *d*-element Y. The “inserted” _39_YF_3_ connects the _67_HoF_3_ and _68_ErF_3_ components of the full QS with continuous lines of liquidus and solidus into the short composite “HoF_3_-YF_3_-ErF_3_” QS.

If the location of dimorphic YF_3_ is in the short composite “HoF_3_-YF_3_-ErF_3_” QS with monomorphic HoF_3_, its ***T***_fus_ = 1155 °C and ***T***_trans_ = 1077 °C [11] should be between that of ErF_3_ (***T***_fus_ = 1146 °C and ***T***_trans_ = 1117 °C [24]). But ***T***_fus_ of YF_3_ is higher and ***T***_trans_ is lower than these of ErF_3_ (Figure 3). Thus, the location of YF_3_ in the phase diagram of the short composite “HoF_3_-YF_3_-ErF_3_” QS cannot be between HoF_3_ and ErF_3_.

### 3.5. Location of YF_3_ Based on the Phase Diagrams of the YF_3_-LnF_3_ Systems in the Short Composite “ErF_3_-YF_3_-TmF_3_” QS at P = P_st_, T > T_st_

**Figure 4:**
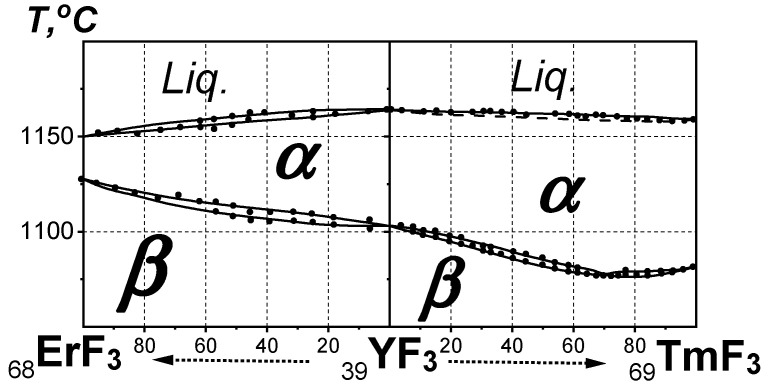
The phase diagram of the short composite “ErF_3_-YF_3_-TmF_3_” QS (***P*** = ***P***_st_, ***T*** > ***T***_st_). Black solid circles correspond to experimental data.

The next possible QS for analyzing the location of YF_3_ is the short composite “ErF_3_-YF_3_-TmF_3_” QS, which is shown in Figure 4. It consists of the ErF_3_-YF_3_ and YF_3_-TmF_3_ systems.

The ***T***_trans_ of YF_3_ (1077 °C) is higher than that of TmF_3_ (1053 °C). At the same time, the ***T***_fus_ values of both fluorides are almost identical (1155 and 1158 °C). They have the same types of structures (***β***- and ***α***-).

The location of YF_3_ in the phase diagram of the short composite “ErF_3_-YF_3_-TmF_3_” QS is between ErF_3_ and TmF_3_. This corresponds to the location of YF_3_ based on its polymorphism and the structure of modifications.

From the literature data concerning the location of YF_3_ in the *Ln*F_3_ series the following statements were obtained:

(1) Structural data are not available for the entire (*R*,*Ln*)F_3_ series. These data are not sufficient for precise analysis of LC and determination of “*pseudo* Z_Y_” of YF_3_.
(2) Structural changes in large arrays of REE compounds of different homologous series with different chemical bonds exhibit uncontrolled shifts in the areas of change in the type of structure along the Z-axis. These data are unsuitable for precise studies of LC.
(3) The thermal and thermochemical properties and phase diagrams are related to the common property—***T***_trans_. This common property determines the location of YF_3_ between ErF_3_ and TmF_3_, which is shifted by Z = 1 compared to the location based on V_form_. The reason for this shift remains unknown.
(4) The phase diagrams of the short composite “HoF_3_-YF_3_-ErF_3_” QS and the particular HoF_3_-ErF_3_ system do not confirm the location of YF_3_ between HoF_3_ and ErF_3_.
(5) In the short composite “ErF_3_-YF_3_-TmF_3_” QS, YF_3_ is located between ErF_3_ and TmF_3_.
(6) To analyze the subtle features of the LC evolution in the *Ln*F_3_ series and obtain *Spec-zd Emp* SIR for (*R*,*Ln*)F_3_, the structural properties of all REE trifluorides obtained under the same technological conditions are required.

### 3.6. Definition of “Pseudo Z_Y_” Using X-ray Diffraction Data

The integer value Z = 39 does not determine the _39_YF_3_ location (“*pseudo* Z_Y_”) in the *Ln*F_3_ series. This can be determined by calculating V**_form_** from the _39_YF_3_ unit cell parameters and obtaining “*pseudo* Z_Y_” by the equation V_form_ = ***f_2_***(Z). “*Pseudo* Z_Y_” corresponds to the exact place of _39_YF_3_ in the *Ln*F_3_ series in terms of its structural properties.

**Figure 5:**
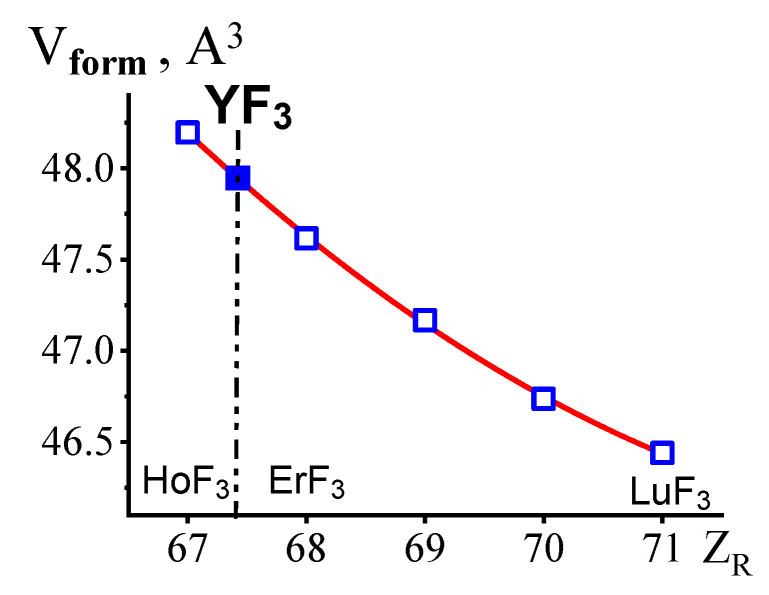
The location of YF_3_ in the *Ln*F_3_ (*Ln* = Ho-Lu) series based on V_form_. Open blue rectangles—V_form_s of ***β***-*Ln*F_3_ (*Ln* = Ho-Lu), the solid blue rectangles correspond to V_form_ of ***β***-YF_3_.

The V_form_s were calculated from the ***β***-YF_3_ and ***β***-*Ln*F_3_ (*Ln* = Ho-Lu) unit cell parameters (Table 2). In Figure 5, the open blue rectangles indicate the change in V_form_ in the ***β***-*Ln*F_3_ (*Ln* = Ho-Lu) series. The V_form_ of ***β***-YF_3_ is indicated by the solid blue rectangle.

The V_form_ = ***f_2_***(Z) curve is well approximated by a second-degree polynomial (red curve in Figure 5):

V_form_ = ***f_2_***(Z) = 0.0419·Z^2^ − 6.22156·Z + 276.9491. (1)

V_form_ = 47.948 Å^3^ of YF_3_ determined from this equation corresponds to “*pseudo* Z_Y_” = 67.42. In the particular HoF_3_-ErF_3_ system this fractional Z value corresponds to the (Ho_0.58_Er_0.42_)F_3_ composition. This is close to the composition of the particular HoF_3_-ErF_3_ system with the morphotropic ***β***- to ***α***- structural change according to the eutectic phase reaction:

Liq ↔ ***β***-(Ho_0.64_Er_0.36_)F_3_ + ***α***-(Ho_0.33_Er_0.66_)F_3_ (2)

The occurrence of “*pseudo* Z_Y_” in the region of the equilibrium existence of ***β***-type solid solutions in the HoF_3_-ErF_3_ system indicates that YF_3_ belongs to the SSGr ***C***. This contradicts the well-studied dimorphism of YF_3_ [27] and its affiliation with the SSGr ***D***.

The differences in the YF_3_ location among *Ln*F_3_ are related to the groups of properties by which this location is determined: structural and thermophysical. It can be assumed that they reflect the contributions of different natures to the total amount of LC.

The separation of the LC contribution (inside the “*Ln* nucleus—4*f* electrons” atomic subsystem) from the *Ln*F_3_ volume changes as a result of polymorphic (morphotropic) changes in the type and density of the structure requires further study and discussion.

The precise determination of the _39_YF_3_ location in the *Ln*F_3_ series from the structural data (V_form_) showed that it is located between HoF_3_ and ErF_3_ with “*pseudo* Z_Y_” = 67.42.

Structural characteristics are used for precise study of LC in the *Ln*F_3_ series and the volume changes caused by it. Therefore, they were chosen as defining the location of YF_3_ between HoF_3_ and ErF_3_.

## 4. Conclusions

LC of 14 *Ln*^3+^ from _58_Ce^3+^ to _71_Lu^3+^ consists of the interaction in the internal “*Ln* nucleus—4*f*-electrons” atomic subsystem. The REEs include Sc, Y, La and 14 *Ln*. They are located in 4–6th periods of the subgroup of group III. The electronic structure of *R* distinguishes the short (*d*- Sc, Y, La) and long (14 *f*-elements *Ln* = Ce-Lu) series.

The first chemical consequence of LC is the generation of the new conglomerate of 16 *R*F_3_ by mixing fluorides of 2 *d*- (Y, La) and 14 *f*-elements. Its creation is based on the structural and chemical proximity of members of the short and long *R*F_3_ series. Thus, LC indirectly determines the location of YF_3_ in the *Ln*F_3_ series.

In the series of REE compounds with different types of chemical bonds, the shift of the yttrium compounds site is known, explicable, and natural. However, according to studies of different *Ln*F_3_ properties, the location of YF_3_ may differ. Such differences can be caused by the differences in chemical bonds in different homologous series and by experimental errors that differ in different methods.

The location of YF_3_ in the *Ln*F_3_ series depends on two groups of properties: structural (unit cell parameters and V_form_s) and thermochemical (temperatures and heats of phase transformation, phase diagrams).

For precise structural characterization, crystals of ***β***-YF_3_ and five ***β***-*Ln*F_3_ (*Ln* = Ho–Lu) were obtained and studied. V_form_s (at ***P***_st_ and ***T***_st_) calculated from the unit cell parameters determine the location of YF_3_ between HoF_3_ and ErF_3_.

According to Δ***H***_fus_ and Δ***H***_trans_, YF_3_ is located in the *Ln*F_3_ dimorphic SSGr ***D*** (*Ln* = Er-Lu). Δ***H***_fus_ and Δ***H***_trans_ cannot provide a more precise position of YF_3_ inside the SSGr ***D*** because they are close to each other.

According to ***T***_trans_, YF_3_ is located between ErF_3_ and TmF_3_. This differs by +1 from its location which was determined based on V_form_s. Simultaneously, this location does not contradict PolTrs and belonging of YF_3_ to the SSGr ***D*** of *Ln*F_3_.

The phase diagrams of the short composite “HoF_3_-YF_3_-ErF_3_” and “ErF_3_-YF_3_-TmF_3_” QS and the particular HoF_3_-ErF_3_ system were applied for the first time to determine the location of YF_3_ in the *Ln*F_3_ series. The phase diagrams are included in the group of thermal properties because they contain ***T***_fus_ and ***T***_trans_. The phase diagrams locate YF_3_ between ErF_3_ and TmF_3_.

To determine the location of YF_3_, the structures of ***β***-YF_3_ and five ***β***-*Ln*F_3_ (*Ln* = Ho-Lu) were studied at 293 K, and their V_form_s were calculated. In the short “from HoF_3_ to LuF_3_” QS using V_form_ = 47.948 Å^3^ the location of YF_3_ was determined between HoF_3_ and ErF_3_ with “*pseudo* Z_Y_” = 67.42.

## Figures and Tables

**Table 1:** Impurity composition of the YF_3_ samples produced by Pyshma, wt. %.

Nd	<0.0005	Dy	0.0002	Si	<0.001	Tm	0.0005
Sm	0.0002	Ho	<0.001	Mn	<0.00005	Fe	0.0005
Gd	0.0005	Cu	<0.0005	Co	<0.00005	Ti	<0.00005
Tb	0.0001	O	270 ppmw	Ni	0.00005	Cr	0.00005

**Table 2:** Experimental crystallographic characteristics and results of the structure refinement for ***β***-YF_3_ and ***β*-***Ln*F_3_ (*Ln* = Ho-Lu) at 293 K (this study).

*R*F_3_	*β*-YF_3_	*β*-HoF_3_	*β*-ErF_3_	*β*-TmF_3_	*β*-YbF_3_	*β*-LuF_3_
ICSD ID	2254323	2254302	2254320	2254321	2254415	2254322
Crystal system	Orthorhombic					
Sp.gr., Z	*Pnma*, 6					
*a* (Å)	6.3666 (2)	6.4055 (2)	6.3500 (5)	6.2792 (3)	6.2168 (3)	6.1437 (4)
*b* (Å)	6.8579 (3)	6.8739 (3)	6.8435 (7)	6.8141 (3)	6.7852 (3)	6.7606 (3)
*c* (Å)	4.3927 (1)	4.3784 (2)	4.3829 (4)	4.4095 (3)	4.4318 (2)	4.4724 (3)
V (Å ^3^)	191.79 (1)	192.78 (1)	190.46 (3)	188.67 (2)	186.94 (2)	185.76 (2)
V_form_	47.948	48.195	47.615	47.168	46.735	46.440
*D_x_* (g·cm^−3^)	5.0528	7.6464	7.8205	7.9538	8.1732	8.2942
μ (mm^−1^)	16.441	21.751	23.486	24.981	26.71	28.387
*T*_min_, *T*_max_	0.0891, 0.1878	0.0594, 0.1564	0.0391, 0.1312	0.0628, 0.1605	0.0222, 0.1051	0.0211, 01031
Shape, color	colorless	light yellow	light rose	colorless	colorless	colorless
Diameter (mm)	0.20					
Wavelength (Å)	0.56087					
Θ range (deg)	4.35–72.65	4.36–72.85	4.36–72.78	4.34–73.03	4.33–72.82	4.31–72.87
Refl. collected	19,376	20,349	20,522	19,403	19,993	19,644
Refl. unique/*R*_int_	3573/4.55	3800/5.14	3342/5.00	3301/5.06	3333/4.65	2895/5.15
Refin. method	Full matrix least squares on *F*					
Param/Restrains	23	38/0	38/0	38/0	38/0	66/0
*R*/*wR*, %	2.26/3.70	2.38/3.93	2.05/3.10	2.60/4.01	2.80/4.11	2.51/3.39
Δρ_min_/Δρ_max_, Å^−3^	−2.70/1.48	−4.48/4.39	−4.29/3.64	−4.54/2.84	−4.60/3.64	−4.26/3.87
GOF	1.36	1.47	1.43	1.40	1.62	1.39

**Table 3:** The Wyckoff positions (W.p.), site occupancy factors (s.o.f.), fractional coordinates, and equivalent thermal displacement parameters of atoms in ***β***-YF_3_ and ***β***-*Ln*F_3_ (*Ln* = Ho-Lu) at 293 K.

*R*F_3_	Ion	W.p.	s.o.f.	*x*/*a*	*y*/*b*	*z*/*c*	U_eq_
YF_3_	Y	4*c*	1	0.367845(12)	¼	0.05981(2)	0.005423(11)
	F(1)	4*c*	1	0.52302(15)	¼	0.5901(2)	0.00976(12)
	F(2)	8*b*	1	0.16454(10)	0.06335(8)	0.37686(16)	0.00856(7)
HoF_3_	Ho	4*c*	1	0.36809(3)	¼	0.06137(4)	0.00483(5)
	F(1)	4*c*	1	0.5217(2)	¼	0.5870(3)	0.00925(16)
	F(2)	8*b*	1	0.16429(16)	0.06318(16)	0.3803(2)	0.00866(10)
ErF_3_	Er	4*c*	1	0.36802(3)	¼	0.06027(4)	0.00521(4)
	F(1)	4*c*	1	0.5231(2)	¼	0.5909(3)	0.00967(14)
	F(2)	8*b*	1	0.16486(14)	0.06248(12)	0.37654(17)	0.00871(9)
TmF_3_	Tm	4*c*	1	0.36725(3)	¼	0.05609(4)	0.00477(4)
	F(1)	4*c*	1	0.5252(2)	¼	0.5971(5)	0.0103(2)
	F(2)	8*b*	1	0.16454(16)	0.06304(15)	0.3716(3)	0.00867(12)
YbF_3_	Yb	4*c*	1	0.36718(3)	¼	0.05395(5)	0.00577(5)
	F(1)	4*c*	1	0.5264(3)	¼	0.6017(4)	0.0114(2)
	F(2)	8*b*	1	0.16510(16)	0.06231(14)	0.3681(2)	0.00897(12)
LuF_3_	Lu	4*c*	1	0.36689(7)	¼	0.05044(10)	0.00759(17)
	F(1)	4*c*	1	0.5278(3)	¼	0.6056(4)	0.0110(2)
	F(2)	8*b*	1	0.16448(18)	0.06265(13)	0.3634(3)	0.00951(11)

**Table 4:** The SSGrs ***A***–***D*** of *R*F_3_.

SSGrs	*R*F_3_	*R*F_3_ Structural Types
***A***	LaF_3_, CeF_3_, PrF_3_, and NdF_3_ monomorphic	***t***-
***B***	PmF_3_, SmF_3_, EuF_3_, and GdF_3_ dimorphic	(1) ***t***- high-temperature (2) ***β***- low-temperature
***C***	TbF_3_, DyF_3_, and HoF_3_ monomorphic	***β***-
***D***	ErF_3_, TmF_3_, YbF_3_, and LuF_3_ dimorphic	(1) ***α***- high-temperature (2) ***β***- low-temperature

**Table 5:** The location of ***β***-YF_3_ in the *Ln*F_3_ series based on V_form_ (literature data).

Reference	Unit Cell Parameters, Å			V_form_ (*T*_st_, *P*_st_)	YF_3_ Location
	*a*	*b*	*c*		
1953 Zalkin [26]	6.353	6.850	4.393	47.79	between HoF_3_ and ErF_3_
1971 Spedding [11]	6.367	6.859	4.394	47.97	between HoF_3_ and ErF_3_
1974 Greis [20]	6.4027	6.8843	4.3980	48.46	between DyF_3_ and HoF_3_

## Data Availability

The data presented in this study are available on request from the corresponding author. The data on the crystal structures are deposited in the Cambridge Structural Database (CSD nums. 2254323, 2254302, 2254320, 2254321, 2254415, 2254322).
